# Simultaneous Formation of a Fully Organic Triply Dynamic Combinatorial Library

**DOI:** 10.1021/acs.orglett.1c01042

**Published:** 2021-04-27

**Authors:** Wojciech Drożdż, Anna Walczak, Artur R. Stefankiewicz

**Affiliations:** † Faculty of Chemistry, Adam Mickiewicz University, Uniwersytetu Poznańskiego 8, 61-614 Poznań, Poland; ‡ Center for Advanced Technologies, Adam Mickiewicz University, Uniwersytetu Poznańskiego 10, 61-614 Poznań, Poland

## Abstract

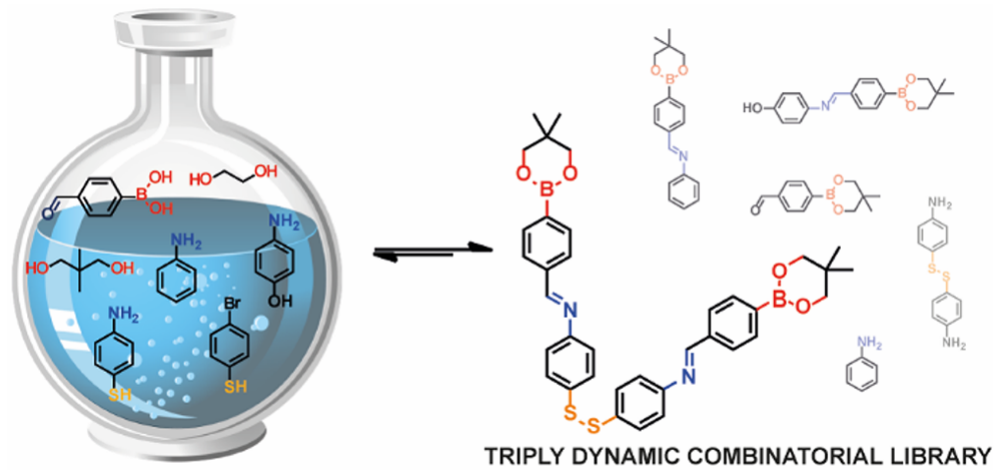

Here we report the simultaneous formation of doubly and triply dynamic libraries as a result of exchange reactions between functionalized organic building blocks. A combination of three different reversible covalent linkages involving a boronate ester transesterification along with an imine and disulfide exchange was employed to generate a new type of fully organic triply dynamic molecular assembly.

Multidynamic systems capable of constitutional interconversions are of increasing interest, as they efficiently implement chemical diversity and lead to “informed” dynamics with applications in the medical and pharmaceutical industries including drug delivery.^1^ Thus, the simultaneous use of several reversible covalent linkages is essential to the expansion of the range of constituents expressed in a Dynamic Combinatorial Library (DCL).^2^ Those most extensively used in combinatorial systems currently are disulfide,^3^ boronate ester,^4^ hydrazone,^5^ and imine^6^ bonds. The combination of more than one reversible bond, within a single library, can lead to a significant increase in both the number of possible products formed and factors that can effect the features of the products such as self-healing, degradability, recyclability, or even shape memory.

It has been reported^7^ that structural diversity within a DCL is increased by using thioester and disulfide exchange processes, generating a doubly dynamic library formed either competitively or orthogonally. In other instances, such DCLs have been obtained using different combinations of disulfide, hydrazone, imines, and boronate ester exchange reactions.^8^ An interesting achievement was the use two of the above-mentioned covalent linkages in the formation of linear small-molecular motors.^9^ Another extraordinary example comes from the Otto group involving an antiparallel dynamic system where two chemistries, thiol–disulfide and thio-Michael exchange, operate simultaneously.^10^

The use of three or more reversible bonds within a dynamic library is quite a challenge due to the complexity of the resulting systems. However, the benefits of a multitude of labile bonds make such architectures more and more popular among scientists. On the one hand, one of the pioneering works on complex orthogonal libraries was presented by Matile, in which the optimized conditions enabling the independent exchange of three orthogonal bonds (disulfide, hydrazone, and boronate ester) was presented.^5,11^ Furlan, on the other hand, investigated DCLs composed of disulfides, thioesters, and hydrazones under conditions ensuring the efficient exchange of the latter, leaving the sulfur moieties intact^2^ and vice versa.^12^ Bonifazi examined multiple-reaction systems based on a disulfide exchange along with boronate and acylhydrazone formations to generate chromophore-supported multicomponent architectures.^13^ Noteworthy is the work of Leclaire, where an appropriate design and the combination of simple building blocks enable the formation of a highly interesting class of compounds capable of capturing CO_2_.^14^ More recently, Anslyn and co-workers reported reversible exchange processes involving four independent linkages, namely, boronic ester, disulfide, hydrazone, and coordination bonds.^15^ Although such examples define the essence of multidynamic systems, those operating simultaneously under the same conditions are still very rare.^14,16^ The examples of multidynamic molecules presented above, although very interesting and sophisticated, contain a hydrazone bond in their structure. The imine group, the reactivity and stability of which is significantly different from the former, is clearly omitted. This may be due to the greater lability of this bond, often resulting in the formation of kinetic products, as opposed to the more stable, thermodynamic hydrazone products.^17^ This prompted us to combine the above-mentioned set of linkages, reliably investigated in terms of reversibility, to generate a fully organic multicomponent combinatorial system.

In this work we report the first example of a solely organic triply dynamic library based on disulfide, boronic ester, and imine bonds operating simultaneously under the same conditions. Although all three distinct linkages are known to exhibit reversibility, they have not been studied within a single reaction system in terms of dynamic combinatorial chemistry (DCC). Thus, all three reversible bonds have been employed for the preparation of covalently assembled triple-level dynamic combinatorial libraries consisting of the following components: *p*-formylphenylboronic acid **1**, two aliphatic dialcohols: ethylene glycol **2** and neopentyl glycol **3**, two aromatic amines: aniline **4** and *p*-hydroxyaniline **5**, and two aromatic thiols: *p*-aminothiophenol **6** and *p*-bromothiophenol **7** (Scheme 1).

**Scheme 1:**
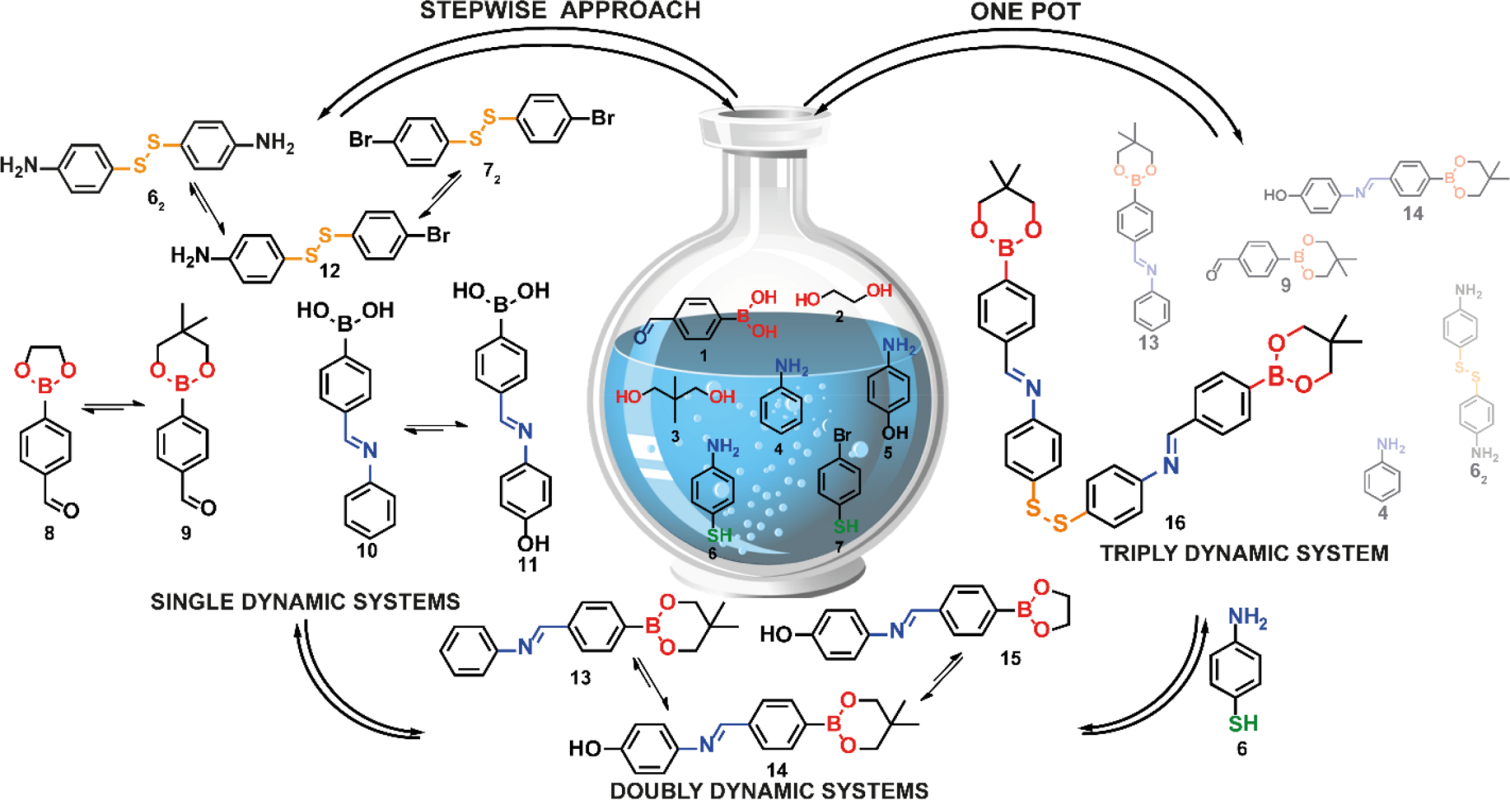
Schematic Formation of Triply Dynamic System Via Two Separate Synthetic Routes—continuous Expansion of the Dynamic Library (Stepwise, from Left to Right) or Simultaneous Formation (One Pot, Right)

To fully investigate the equilibration and final composition of the generated dynamic libraries, all experiments were monitored and analyzed via ^1^H NMR spectroscopy. At least two exchange pathways are possible for each investigated reaction: boronate ester exchange might occur through either hydrolysis/re-esterification (dissociative exchange) or transesterification (associative exchange) mechanisms; imine exchange could proceed through an amine addition to give an aminal intermediate, which then undergoes elimination, or it could involve a metathesis reaction between two imines (unobservable in the present systems, where a single aldehyde was involved), while disulfide exchange could occur through a reaction with a thiol/thiolate or through a metathesis reaction. While for the formation of dynamic systems it is important that the reactions proceed and equilibrate reasonably quickly, regardless of their mechanisms, note that we have found mild reaction conditions so that all three above-mentioned reversible processes could proceed essentially simultaneously. It is also worth emphasizing that all library components employed in this study had good solubility under the conditions used. Thus, the following reaction conditions were employed in all experiments described below: (1) 5 mM of the individual substrate concentration, (2) temperature adjusted to 50 °C, (3) deuterated dimethyl sulfoxide (DMSO-*d*_6_) was used as the reaction medium and also as a mild oxidant (thiol to disulfide oxidation).^18^ Unless otherwise mentioned, reactions were performed for 24 h or until thermodynamic equilibrium was obtained.

Initially, the reversibility and exchange reactions of each of the dynamic bonds were examined separately under the above conditions. The aim of these studies was to evaluate the effectiveness of a specific dynamic bond formation under the condition applied, but it also allowed a comparison of the relative reactivity of the chosen molecular components. First, we investigated the formation of boronic esters from 1 and the two aliphatic diols differing in that the hydroxyl groups were in 1,2 (**2**) or 1,3 (**3**) positions, so that esters formed contained either a five- or a six-membered ring, **8** or **9**, respectively (Figure 1a). Although both ester products were formed, only 30% of **8** was generated, while the formation of **9** occurred in almost quantitative yield (92%), confirming its greater thermodynamic stability.^19^ Preferential formation of a specific boronic ester product was also observed in the two subsequent reactions performed for this system, that is, self-selection and exchange processes, in which molecule **9** was obtained in 81% and 72% yields, respectively (Figure 1a,b). The remaining material consisted of ester **8** (10% and 6% yields, respectively) and unreacted substrates. The second reversible reaction investigated was that of imine formation between aldehyde **1** and aniline **4** and its *p*-hydroxy substituted analogue **5**. After 24 h, the library was analyzed by ^1^H NMR spectroscopy and indicated the generation of the corresponding imine products **10** and **11** in ∼77% and 89% yields, respectively (Figures S5a and S6 in the Supporting Information). As with the boronic esters, self-sorting and exchange reactions were also conducted, clearly showing a preferential formation of **11** compared to **10** (ratios of 76/24 and 69/31, respectively, Figures S5 and S7 in the Supporting Information). This may be due to the greater nucleophilicity of the amine **5** containing an electron-donating OH group in the para position.

**Figure 1:**
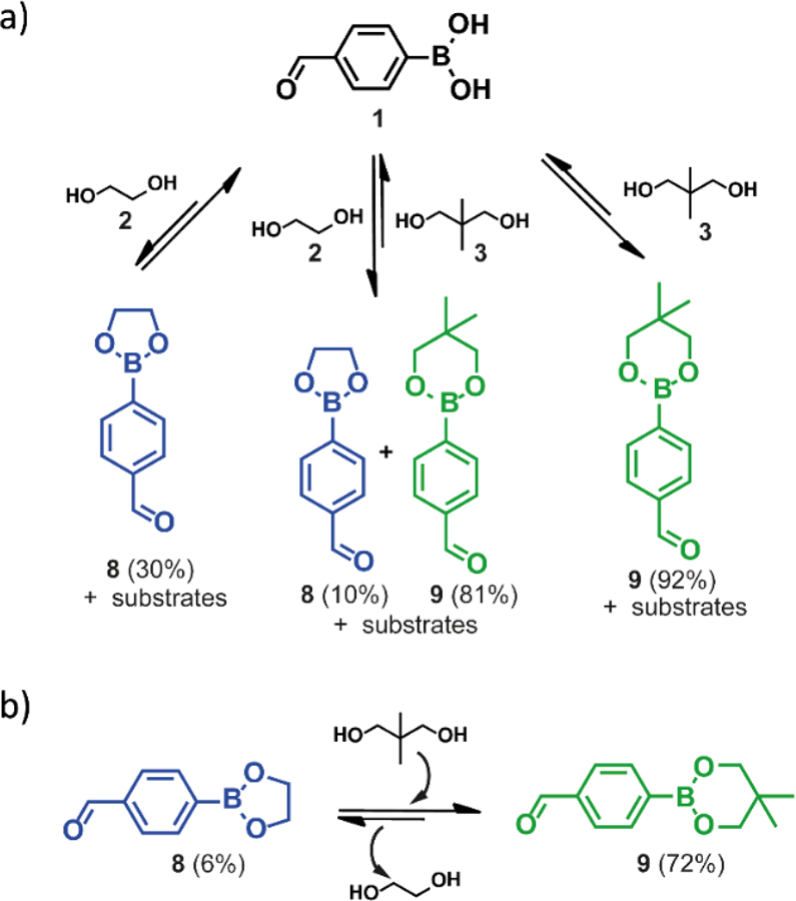
Schematic representation of the experiments performed to establish the thermodynamic equilibrium of boronic esters **8** and **9** formation through (a) self-sorting of starting materials and (b) components exchange between isolated ester **8** and diol **3** (the unreacted materials constituted 22% of the postreaction mixture).

The last linkage studied within a single-level dynamic library was that of the disulfide bond. Unlike the two previously discussed reversible bonds, a symmetrical disulfide formation may result from the autoxidation of an individual reaction component. Thus, the rate of the autoxidation process was monitored by ^1^H NMR spectroscopy, separately for each thiol building block (**6** and **7**), which differ in the type of substituent in the para position. While the signal from the *p*-bromothiophenol **7** disappeared within 9 h as a result of **7**_**2**_ formation, component **6** (*p*-aminothiophenol) required 28 h for the complete oxidation to homodimer **6**_**2**_, indicating a major influence of the thiol structural features on the rate of disulfide formation. (Figures S8–S10 in the Supporting Information). To assess that the disulfide exchange operates under thermodynamic control, two distinct pathways for generating the DCL were explored. In the first, the DCL was engendered by mixing equimolar amounts of thiol components **6** and **7**, while in the second pathway the preoxidized disulfides **6**_**2**_ and **7**_**2**_ were mixed together (Figure S15). In the case of a thermodynamically controlled system, the composition of the generated DCL (a mixture consisting of heterodimer (**12**) and two homodimers (**6**_**2**_ + **7**_**2**_) was expected) should be identical, regardless of the pathway used. The first DCL was prepared by dissolving **6** and **7** (each at 5 mM concentration) in DMSO-*d*_6_ at a temperature adjusted to 50 °C. The library was stirred for 24 h in air in a capped NMR tube to allow oxidation of the thiol building blocks. An analysis of the ^1^H NMR spectrum of the reaction mixture (Figure S15b, top) revealed a fully oxidized library with several sets of doublets corresponding to the aromatic protons of homodimeric products **6**_**2**_ (marked red) and **7**_**2**_ (marked blue), respectively (each accounted for 30% of the library material). The remaining set of peaks was assigned to the heterodimeric disulfide compound **12** (marked green), the predominant species in the mixture (40% of the library material). An essentially identical product distribution (Figure S15b, bottom) was observed in the DCL generated by the alternative pathway (starting from preformed homodimers **6**_**2**_ and **7**_**2**_, each at 5 mM), thus confirming the thermodynamic nature of the investigated equilibria.

Following this characterization of the single-level dynamic libraries, we moved toward a more complex system, where the simultaneous formation and exchange processes within double-level dynamic systems were investigated. These experiments were intended to provide compounds containing both boronic ester and imine dynamic linkages within a single molecular component. For this purpose, two separate DCLs were prepared that, depending on the type of the studied exchange reaction (imine exchange and/or boronic transesterification), differed in the composition of the components used. The first doubly dynamic library was obtained using a stoichiometric mixture of boronic acid **1**, diol **3**, and two different amines, **4** and **5**. The generated DCL was analyzed after 24 h by ^1^H NMR spectroscopy and revealed the presence of two doubly dynamic compounds **13** (marked blue) and **14** (marked red) in the ratio 24/76 along with unreacted substrate (Figure S11 in the Supporting Information). A similar library composition was observed upon the addition of amine **5** to the solution of preformed molecule **13**, which induced the imine exchange reaction, yet left the ester bond intact.

The same procedure was exploited in the second DCL, involving a transesterification in the vicinity of the imine bond. Analogously to the previous experiment, diols **2** and **3** were mixed with boronic acid **1** and *p*-hydroxyaniline **5**. The ^1^H NMR spectrum showed a strong preference toward the formation of product **14** possessing the more stable six-membered boronate ester ring (88% of the products library) compared to its structural analogue **15** (12% of the products library, Figure S12 in the Supporting Information). Subsequently, a transesterification between **3** and isolated **15** was found to give a DCL with the same distribution. These observations highlight again the influence of the structural features of the molecular building blocks on the ultimate constitution of the generated DCLs. The dynamic processes in the second system proceeded orthogonally without the decomposition of a reversible bond not involved in the exchange reaction.

The final step of the present work involved the investigation of a triply dynamic library within the same reaction flask. Although the formation of disulfide, boronate ester, and hydrazone exchange has been previously established for multicomponent surface architectures,^5,11^ a reaction set consisting of the first two linkages and an imine has not been studied. Two approaches were used to evaluate the possibility of simultaneous formation of the three dynamic bonds under the conditions applied. First, the “one pot” approach was performed, where building blocks **1**–**6** (each at 5 mM) were combined together in an equimolar ratio in DMSO-*d*_6_ at a temperature adjusted to 50 °C. After 24 h, the resulting mixture was analyzed by ^1^H NMR spectroscopy (Figure S13 in the Supporting Information), which confirmed the presence of a complex dynamic library of structurally distinct compounds, among which the desired triply dynamic compound **16** was found in trace amounts. In an attempt to reduce the library complexity, which would in turn allow for an easier isolation and a full characterization of **16**, a stepwise approach was performed using only those components that constitute the desired molecule, that is, **1**, **3**, and **6** (each at 5 mM). Although the DCL generated from these building blocks remained complex in solution (Figure S14 in the Supporting Information), it allowed the isolation of **16**, which precipitated out of the reaction mixture in the form of a yellow powder, in 15% yield.

Given its apparently low solubility, one obvious way to promote the formation of this species at the expense of other, more soluble coproducts, is to increase the concentration of DCL components. To our delight, the isolated yield of **16** was significantly enhanced (to 57%) by a 10-fold increase of the initial building block concentration (50 mM). When components **1**, **3**, and **6** were combined under these conditions, a gradual precipitation of a yellow, crystalline solid was observed, which, after filtration and washing with Et_2_O, could be isolated in quantities sufficient for its full characterization. Confirmation of the expected structure of triply dynamic molecule **16** was provided by solution NMR measurements (Figure 2 for ^1^H and Figure S2 in the Supporting Information for ^13^C), mass spectrometry (MS) (Figure S3), and elemental analysis (see Synthetic procedure in the Supporting Information). The ^1^H NMR spectrum shows a well-resolved set of signals with a characteristic imine proton peak at 8.45 ppm (H^5^) and two sharp singlets from −CH_2_ (H^2^) and −CH_3_ (H^1^) moieties, at 3.79 and 1.04 ppm, respectively (Figure 2). The mass spectrum showed an [M + H]^+^ ion, *m*/*z* = 649.2489 and confirmed the expected composition.

**Figure 2:**
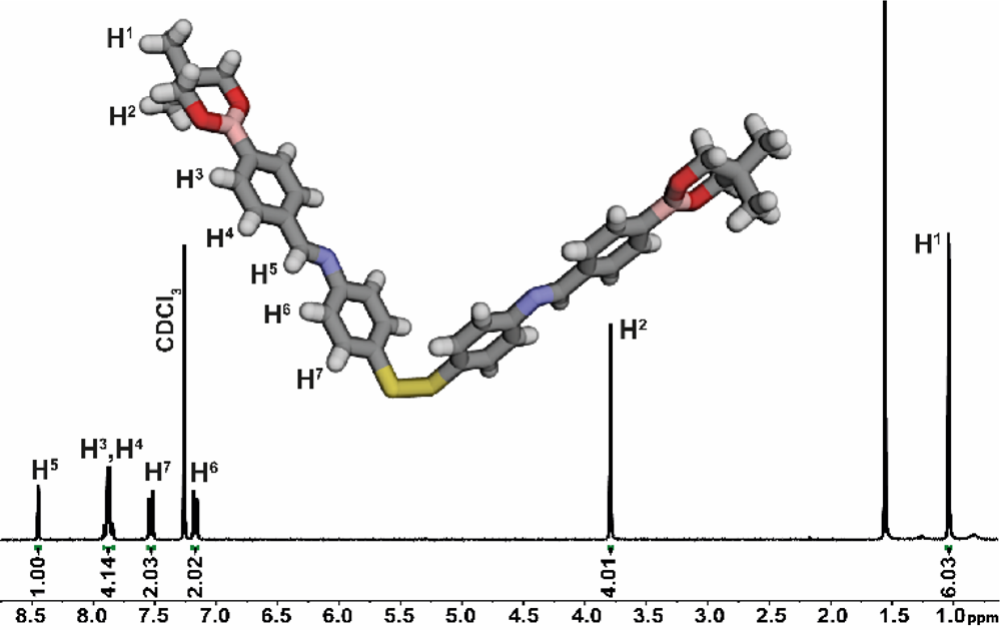
^1^H NMR (300 MHz, CDCl_3_) spectrum of the isolated triply dynamic molecule **16** and (inset) its crystal structure.

The final structural confirmation for **16** was provided by a single-crystal X-ray crystallographic analysis. The molecules are linked by weak CH···π interactions between a methylene hydrogen atom and the aromatic six-membered ring (CH···πPh 2.712(1) Å, Figure S16 in the Supporting Information), and S···CHPh (2.903(1) Å, Figure S17 in the Supporting Information) along the *a*-axis, forming a two-dimensional network.

In conclusion, we have described the first multicomponent reaction system operating simultaneously in a DMSO solution at a slightly elevated temperature and consisting of three distinct reversible linkages, that is, disulfide, boronate, and imine. The gradual increase of the system complexity has ultimately led to the generation of a unique example of a fully organic triply dynamic molecular compound formed in a one-pot reaction between six components via three independent reversible reactions. Such a combination of covalent bonds has never been employed in the formation of a multidynamic molecule. Interestingly, we successfully isolated and fully characterized the final product both in solution and in the solid state, providing the first example of the crystal structure of a molecular compound containing three distinct dynamic linkages. The developed methodology and an analytical protocol put forward in this paper is straightforward and should be widely applicable in the generation of other multidynamic functional architectures possessing more complex topologies such as cages, knots, or polymers. The physicochemical properties and potential functions of such architectures, for instance, in catalysis, to prevent product inhibition or in medicine, to release a drug at a specific site, could then be controlled by a strategic application of a precisely selected exchange chemical reaction.

## Supporting Information Available

Experimental procedures, characterization data, and copies of the NMR spectra, crystallographic data (PDF)

## Accession Codes

CCDC 2057952 contains the supplementary crystallographic data for this paper. These data can be obtained free of charge via www.ccdc.cam.ac.uk/data_request/cif, or by emailing data_request@ccdc.cam.ac.uk, or by contacting The Cambridge Crystallographic Data Centre, 12 Union Road, Cambridge CB2 1EZ, UK; fax: +44 1223 336033.

## Dedication

Dedicated to Prof. Janusz Jurczak on the occasion of his 80th birthday.
