# Supplementary material for: Simultaneous Formation of a Fully Organic Triply Dynamic Combinatorial Library
Source: Org Lett. 2021 Apr 27;23(9):3641–5. doi: 10.1021/acs.orglett.1c01042 (PMC8289287; doi:10.1021/acs.orglett.1c01042)
Supplement: Supplementary file 1 — ol1c01042_si_001.pdf [file ol1c01042_si_001.pdf]

SUPPORTING INFORMATION

Simultaneous Formation of a Fully Organic Triply-Dynamic Combinatorial Library

W. Drożdż,[a,b] A. Walczak [a,b] and A. R. Stefankiewicz [a,b]

[a] Faculty of Chemistry, Adam Mickiewicz University, Uniwersytetu Poznańskiego 8, 61-614 Poznań, Poland.
[b] Centre for Advanced Technologies, Adam Mickiewicz University, Uniwersytetu Poznańskiego 10, 61-614 Poznań, Poland
e-mail: ars@amu.edu.pl

I Materials and methods 2

II Synthetic procedure 2

III Experimental data 6

IV XRD Data 16

I Materials and methods

Solvents and reagents, unless otherwise stated, were purchased from commercial suppliers (Sigma Aldrich, Fluorochem, Across Organics) and used without further purification

NMR. ^{1}H NMR, ^{13}C NMR spectra were recorded at 300 MHz for ^{1}H and 75 MHz for ^{13}C (Bruker Fourier) in deuterated DMSO-d_6 and $CDCl_3$. Chemical shifts are reported in ppm relative to the residual solvent peak. Data are reported as follows: chemical shift (δ), multiplicity (s for singlet, d for doublet), coupling constant (*J* in Hertz), and integration.

Mass Spectrometry. ESI-MS measurements were carried out on AB Sciex (QTOF 5600+) spectrometer.

X-ray diffraction The structural studies for the compound **16** was performed on a New Xcalibur EosS2 diffractometer equipped with a CCD detector. X-ray data were collected at 295 K using graphite-monochromated Mo Kα radiation source ($\lambda\alpha$ = 0.71073 Å) with the ω-scan technique. For data reduction, UB-matrix determination and absorption correction CrysAlisPro[1] software was used. Using Olex2, [2] the structures were solved by direct methods with ShelXT[3] and refined by full-matrix least-squares against F^2 with the program SHELXL[4] refinement package based on the Least Squares minimization. All non-hydrogen atoms were refined anisotropically. Carbon-bound hydrogen atoms were calculated at ideal positions by molecular geometry and refined as rigid groups with U_{iso}(H) = $1.2\ U_{eq}(C)$. Selected structural parameters are reported in Table S1. The data have been deposited in the Cambridge Crystallographic Data Collection (CCDC), deposition numbers CCDC 2057952. These data can be obtained free of charge *via* www.ccdc.cam.ac.uk/data_request/cif, or by emailing data_request@ccdc.cam.ac.uk, or by contacting The Cambridge Crystallographic Data Centre, 12, Union Road, Cambridge CB2.

II Synthetic procedure

Unless otherwise stated, all equilibrium reactions were carried out in an NMR tube in DMSO-d_6, heated in oil bath at 50 °C for 24 h. The concentration of the individual components of the reaction was set up at 5 mM. All NMR spectra were measured at 25 °C.

Compound **16**. *p*-formylphenylboronic acid **1** (33.66 mg, 0.225 mmol, 1 equiv), neopentyl glycol **3** (23.38 mg, 0.225 mmol, 1 equiv), and *p*-aminothiophenol **6** (28.11 mg, 0.225 mmol, 1 equiv), were placed in NMR tube and dissolved in 1 mL of DMSO-d_6. Reaction was heated at 50 °C for 48 hours. Yellow crystalline precipitate obtained in the tube was centrifuged and dried under vacuum for 2 days providing crystalline yellowish solid with the 57% yield (41.49 mg). Suitable crystals were prepared by slow cooling of a hot DMSO-d_6 solution in an NMR tube. ^{1}H NMR (300 MHz, Chloroform-*d*) δ 8.45 (s, 2H, H^5), 7.93 – 7.83 (m, 8H, H^3+H^4), 7.55 – 7.52 (d, ^{2}J = 8.4 Hz, 4H, H^7), 7.18 – 7.16 (d, ^{2}J = 8.4 Hz, 4H, H^6), 3.79 (s, 8H, H^2), 1.04 (s, 12H, H^1). ^{13}C NMR (75 MHz, $CDCl_3$) δ 161.0, 151.7, 137.9, 134.5, 134.3, 129.8, 128.1, 121.8, 77.1, 72.5, 32.0, 22.0; ESI-MS

m/z: $[M+H]^+$ Calcd for $C_{36}H_{39}B_2N_2O_4S_2$ 649.2544; Found: 649.2489. Anal. Calcd for $C_{36}H_{38}B_2N_2O_4S_2$: C, 66.68; H, 5.91; N, 4.32; found: C, 66.52; H, 5.97; N 4.21.

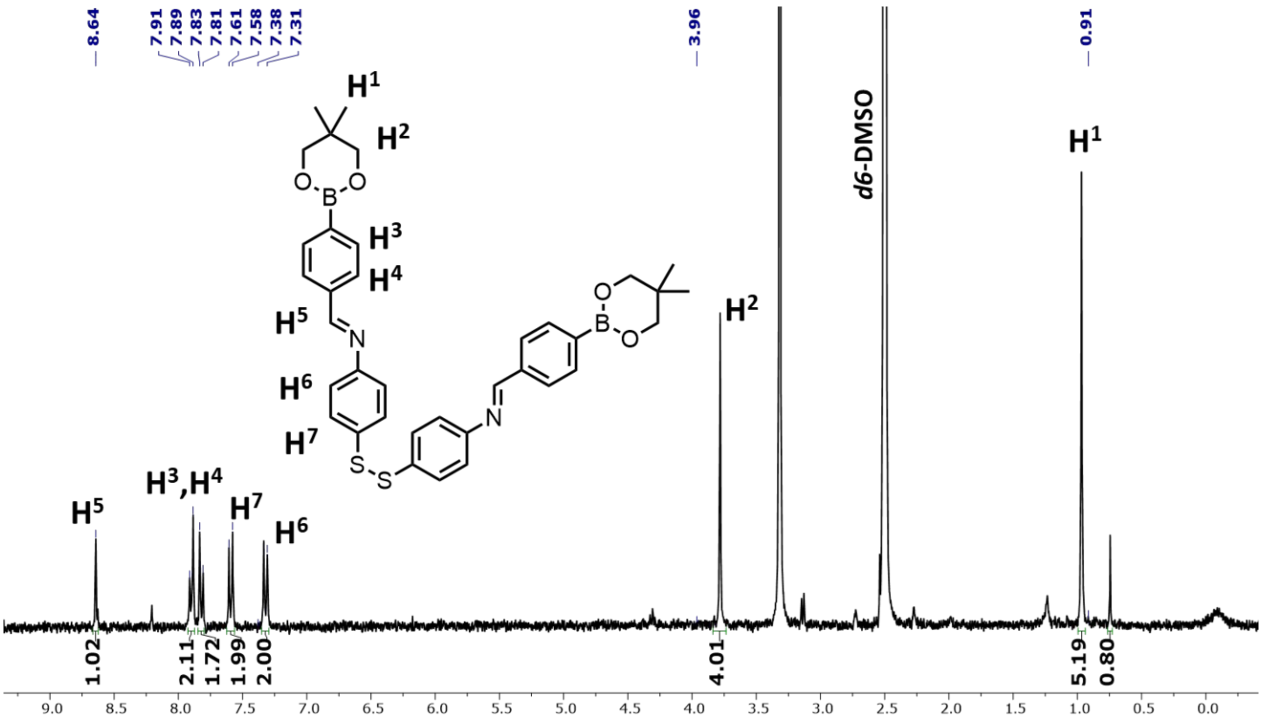

Figure S1. ^{1}H NMR spectrum (300 MHz, DMSO-d_6, temperature: 25 ˚C) of compound **16.**

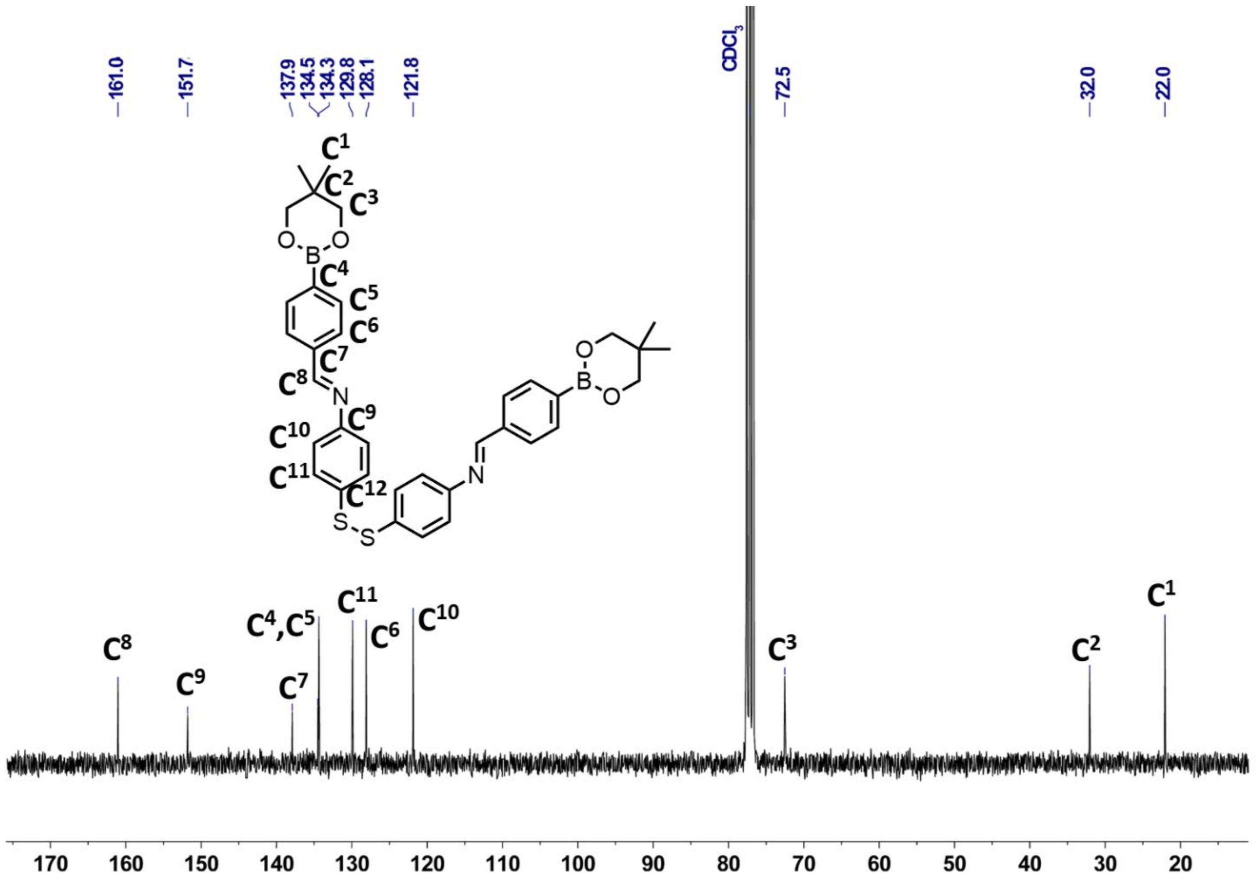

Figure S2. ^{13}C NMR spectrum (75 MHz, $CDCl_3$, temperature: 25 ˚C) of compound **16.**

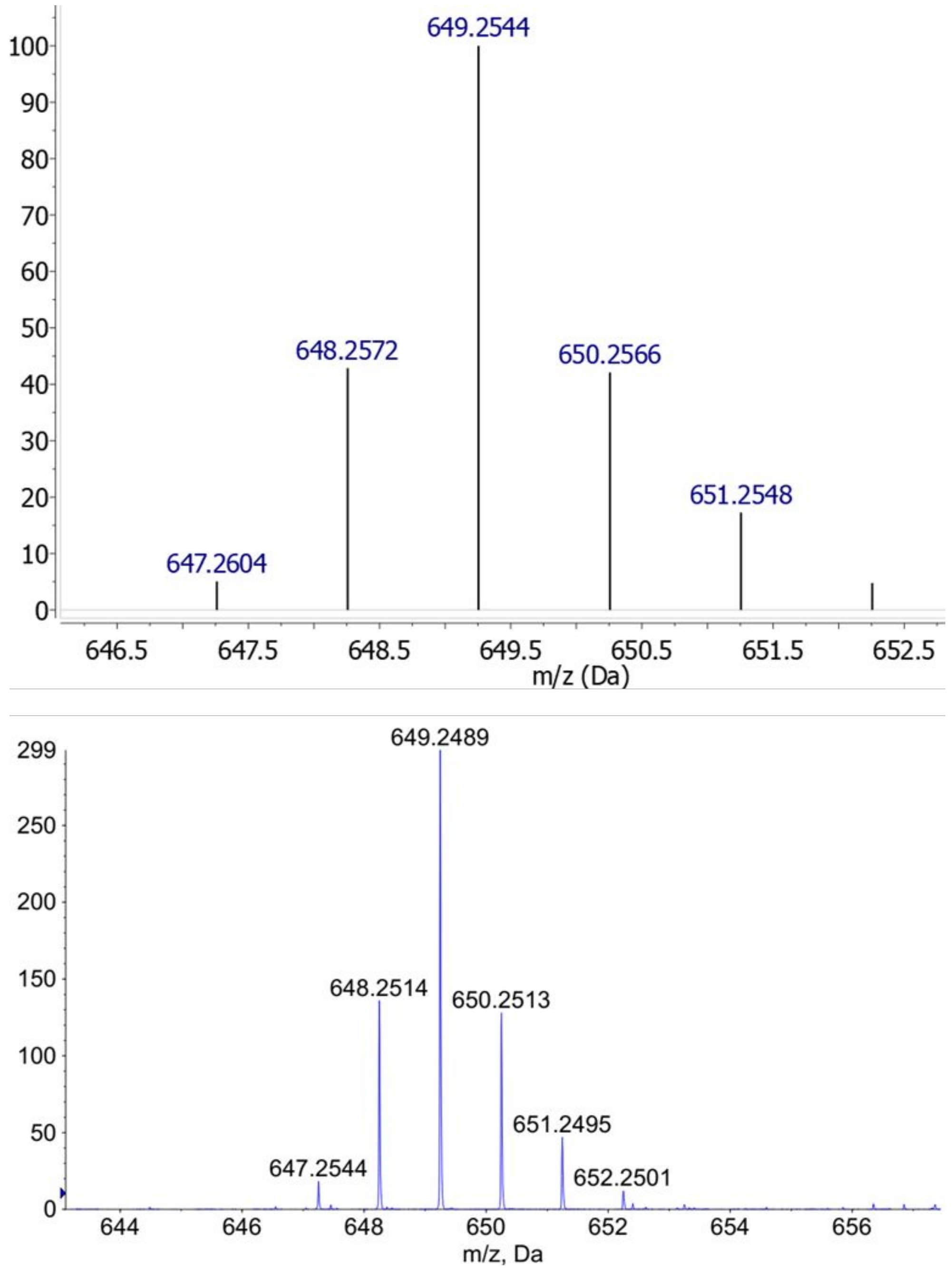

Figure S3. ESI-MS analysis of compound **16**, showing the observed data (bottom) and theoretical isotope model (top).

III Experimental data

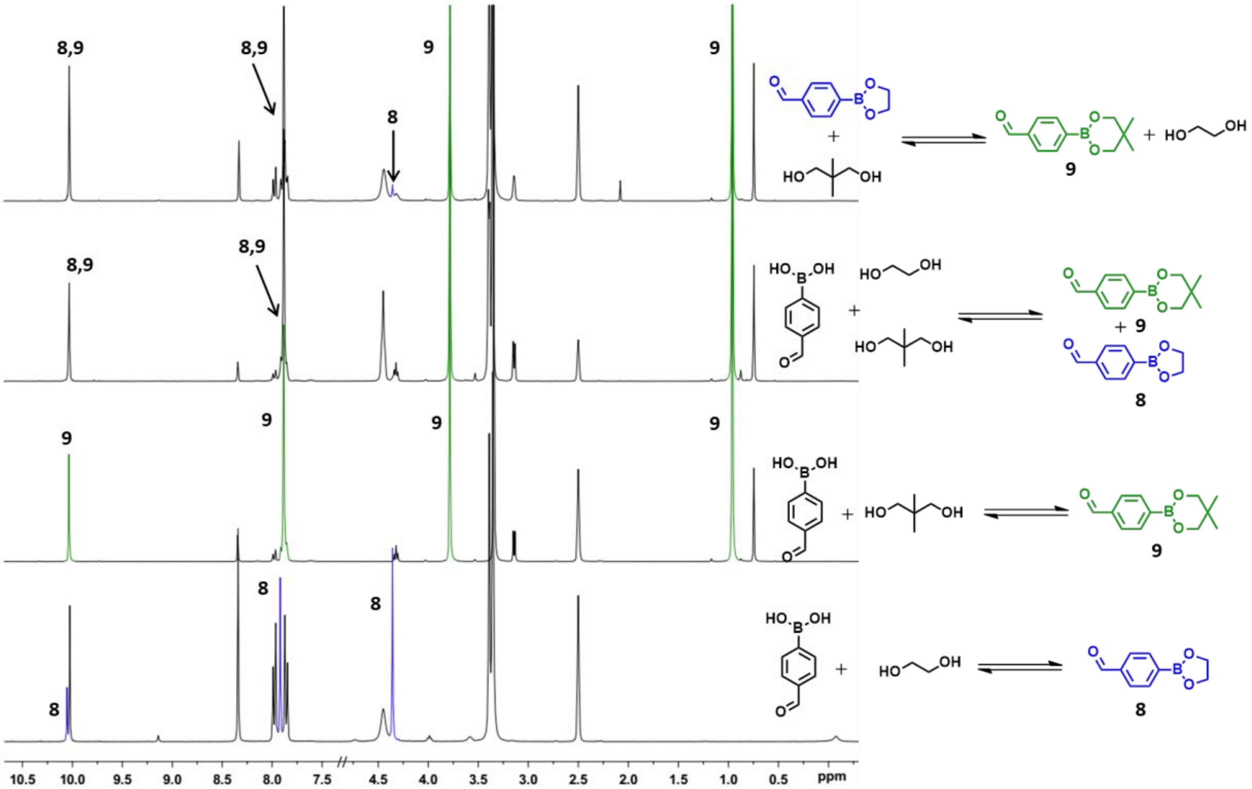

Figure S4. ^{1}H NMR (300 MHz, DMSO-d_6, temperature: 25 ˚C) of boronic ester exchange. All reaction components: 5 mM.

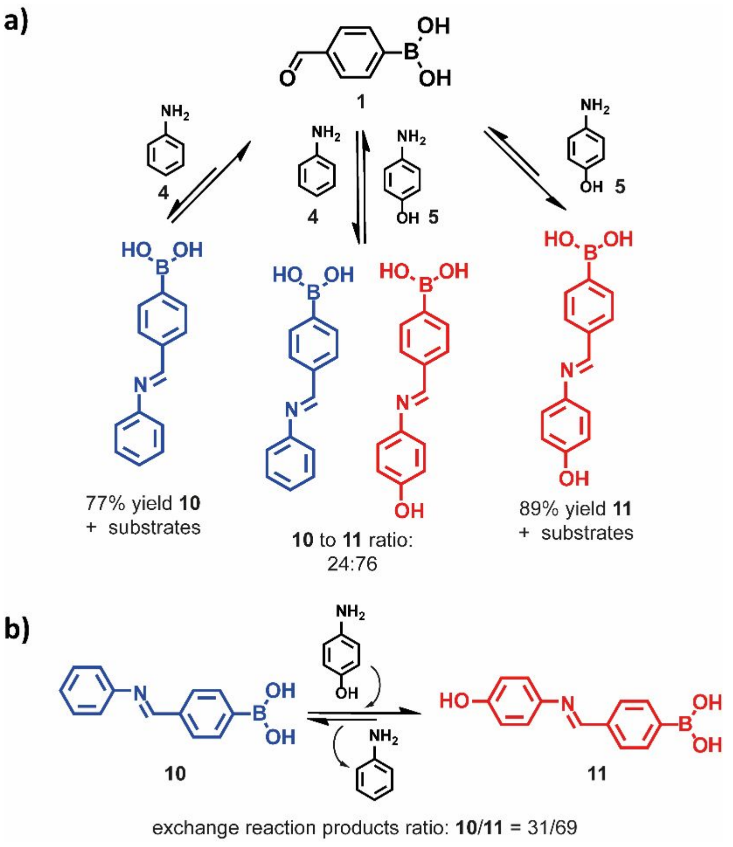

Figure S5. Reaction scheme of the experiments used to establish the thermodynamic equilibrium.

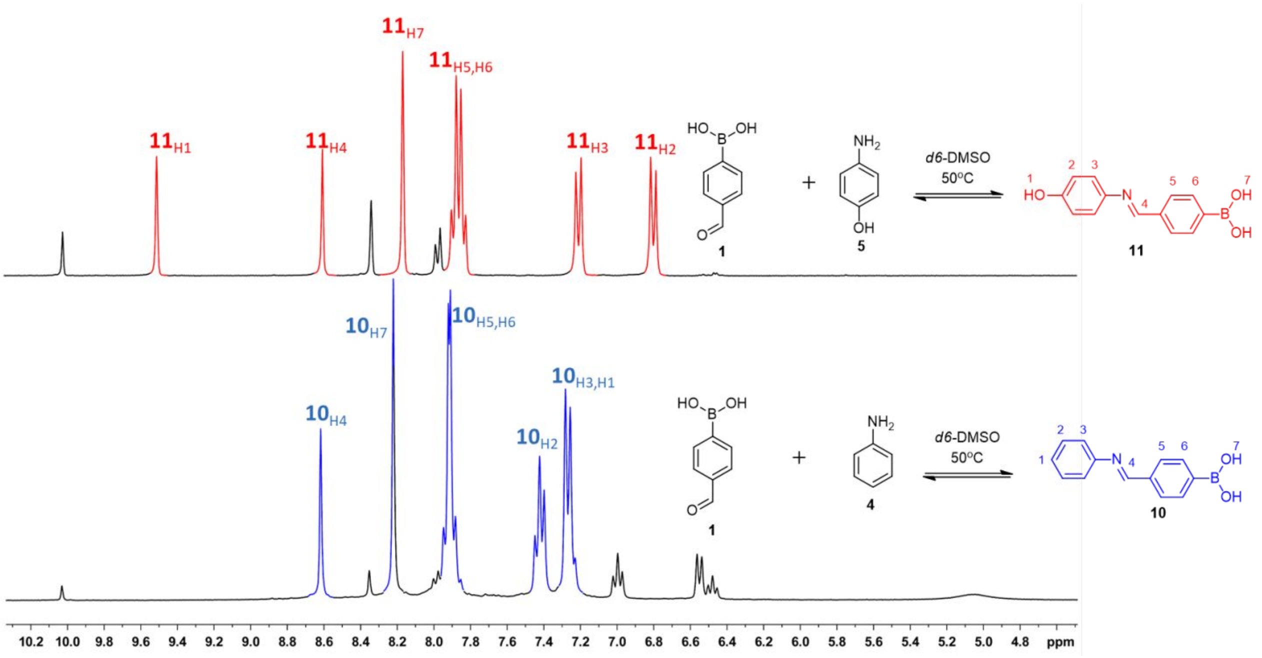

Figure S6. ^{1}H NMR (300 MHz, DMSO-d_6, temperature: 25 ˚C) of single imine formation. All reaction components: 5 mM.

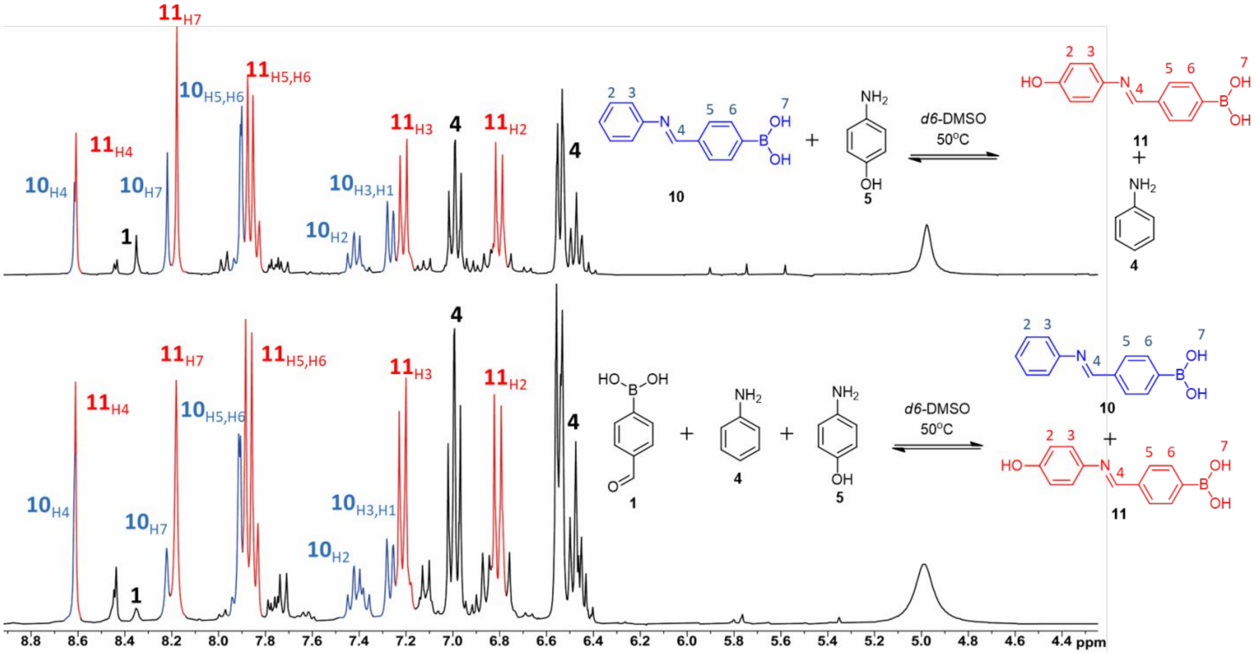

Figure S7. ^{1}H NMR (300 MHz, DMSO-d_6, temperature: 25 ˚C) of thermodynamic library of imine compounds formation through exchange reaction (top) or self-sorting (bottom). All reaction components: 5 mM.

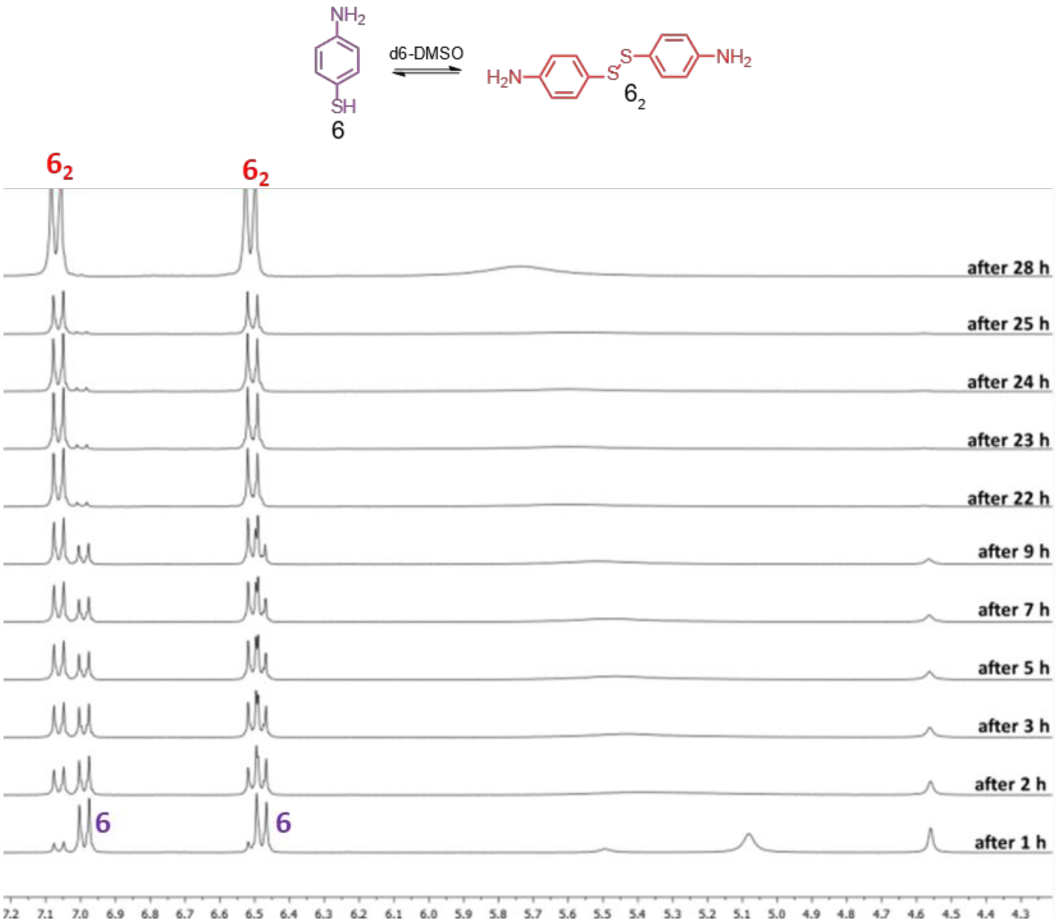

Figure S8. ^{1}H NMR (300 MHz, DMSO-d_6, temperature: 25 ˚C) spectrum of *p*-aminothiophenol **6** self-oxidation. Reaction component: 5 mM.

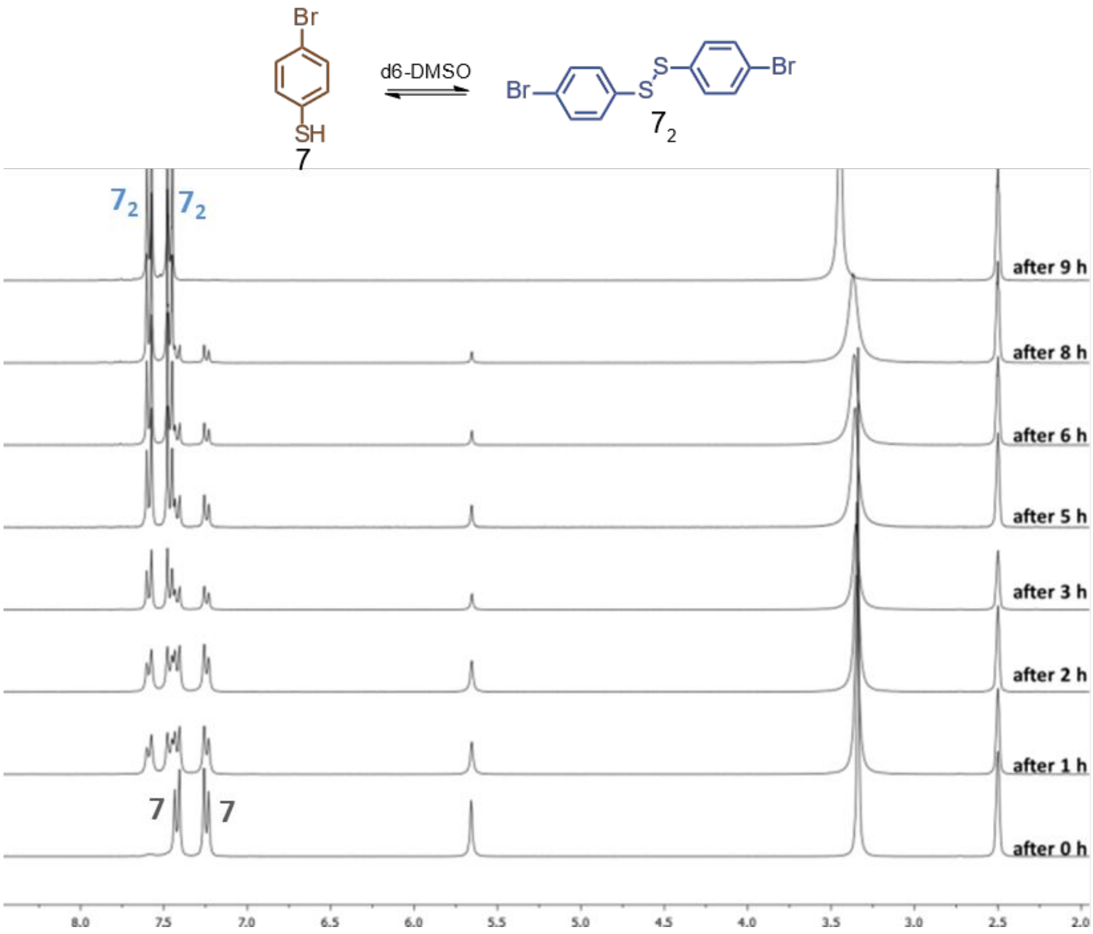

Figure S9. ^{1}H NMR (300 MHz, DMSO-d_6, temperature: 25 ˚C) spectrum of *p*-bromothiophenol **7** self-oxidation. Reaction component: 5 mM.

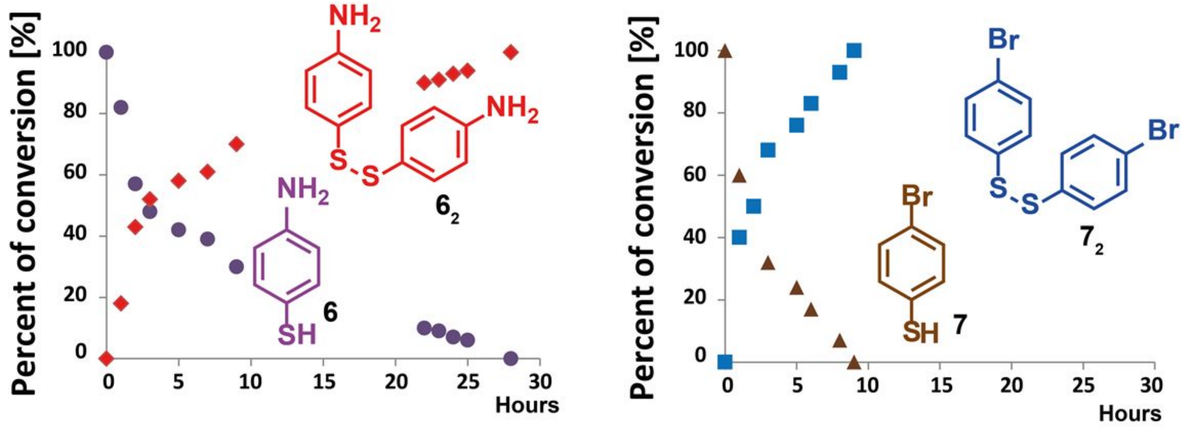

Figure S10. Material-distribution chart of disappearance of thiol component upon oxidation to its disulfide: **left** purple: **6**, red: **6_2**, **right** brown: **7**, blue: **7_2**. All reactions components: 5 mM.

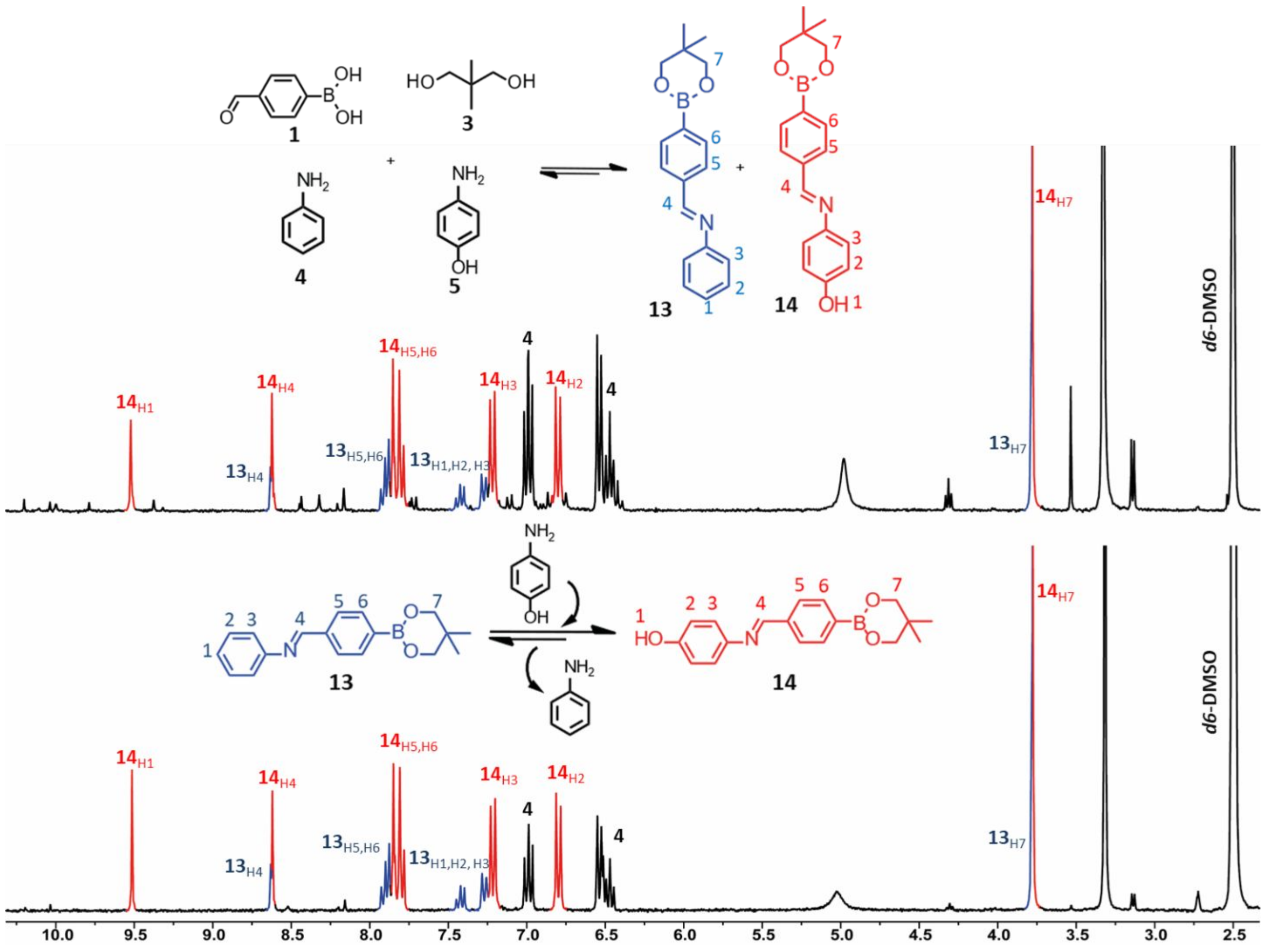

Figure S11. ^{1}H NMR (300 MHz, DMSO-d_6, temperature: 25 °C) spectra showing diagnostic peaks of **13** (blue peaks) and **14** (red peaks) as major products of self-sorting (*top*) and exchange processes (*bottom*). All reaction components: 5 mM.

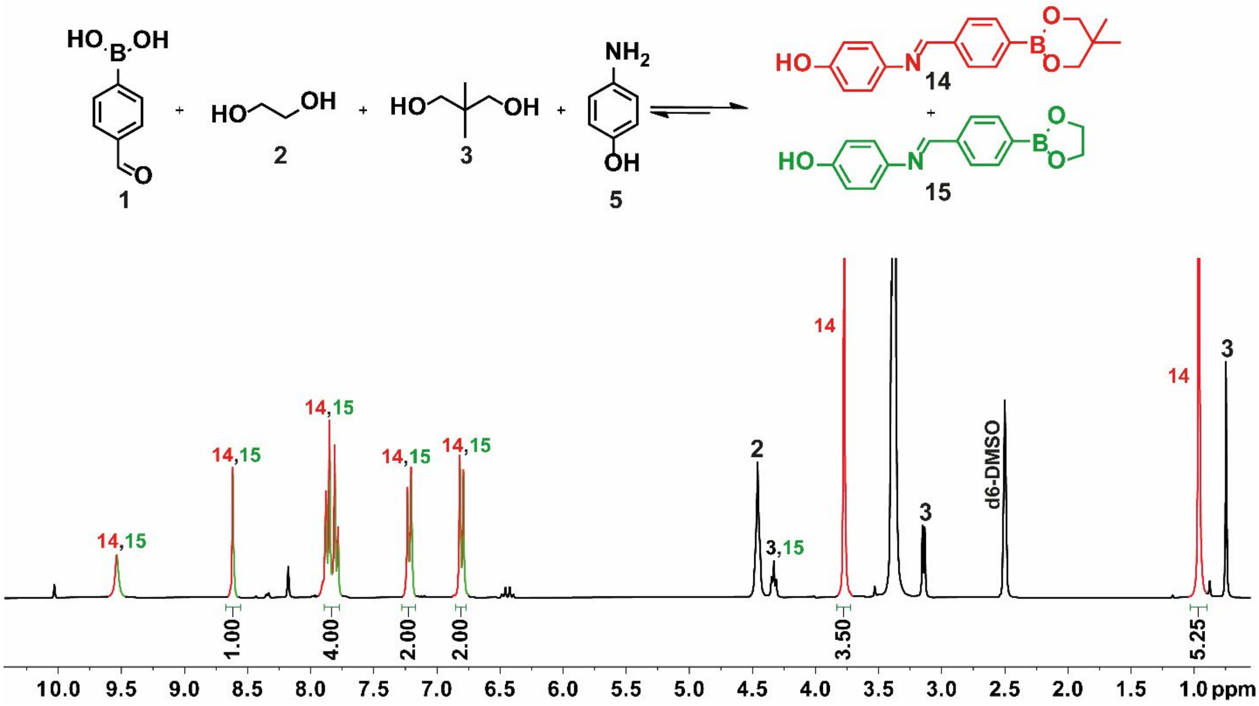

Figure S12. Reaction scheme of the self-sorting process in doubly-dynamic system and ^{1}H NMR (300 MHz, DMSO-d_6, temperature: 25 ˚C) of the generated library. All reaction components: 5 mM.

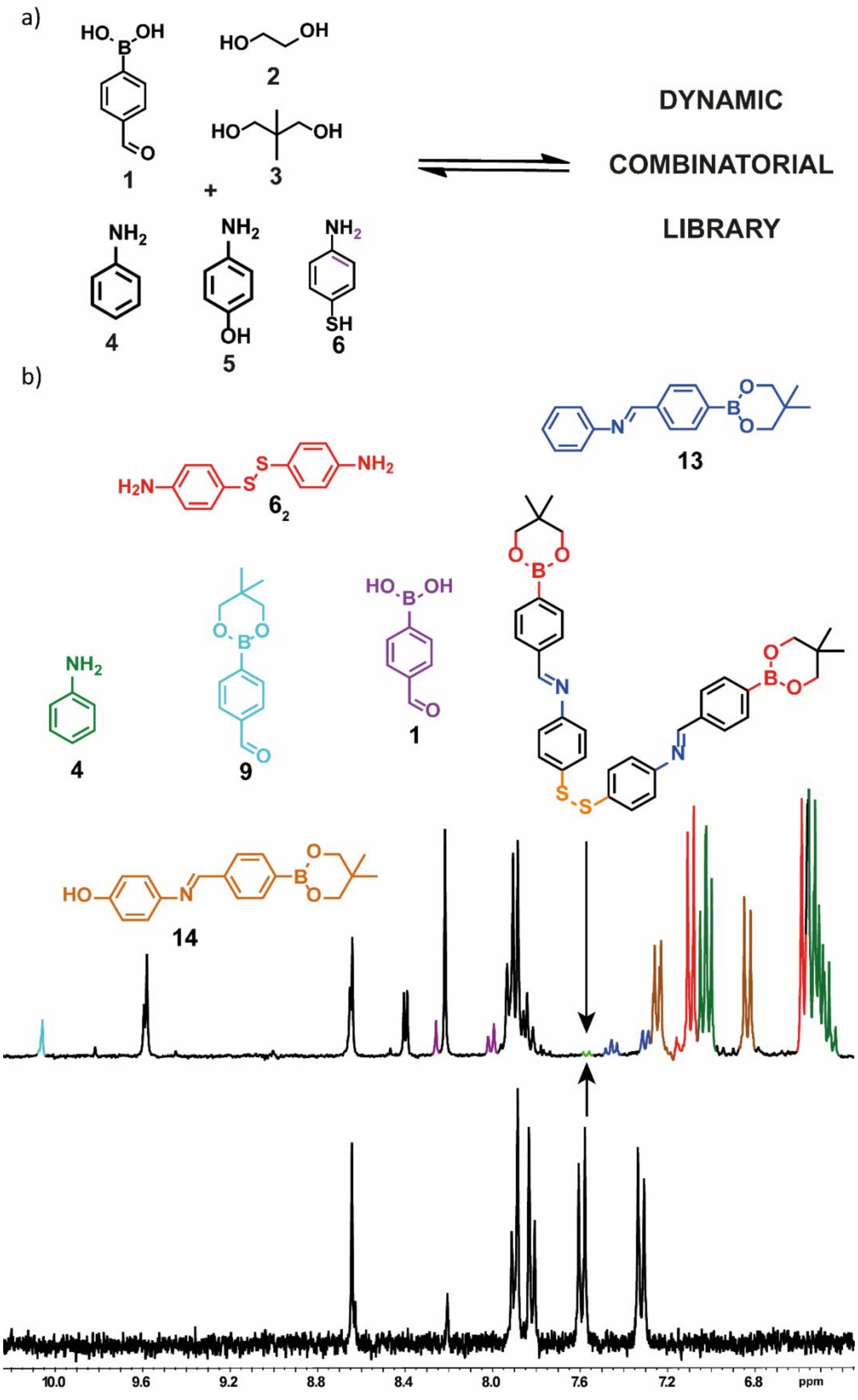

Figure S13. Reaction scheme (a) and ^{1}H NMR (300 MHz, DMSO-d_6, temperature: 25 ˚C) stacked spectra (b) of dynamic library composed from **1**, **2**, **3**, **4**, **5** and **6** starting materials (*top*) and isolated compound **16** (*bottom*). Major products assigned to the corresponding signals on the NMR spectrum with the appropriate colours. All reaction components: 5 mM.

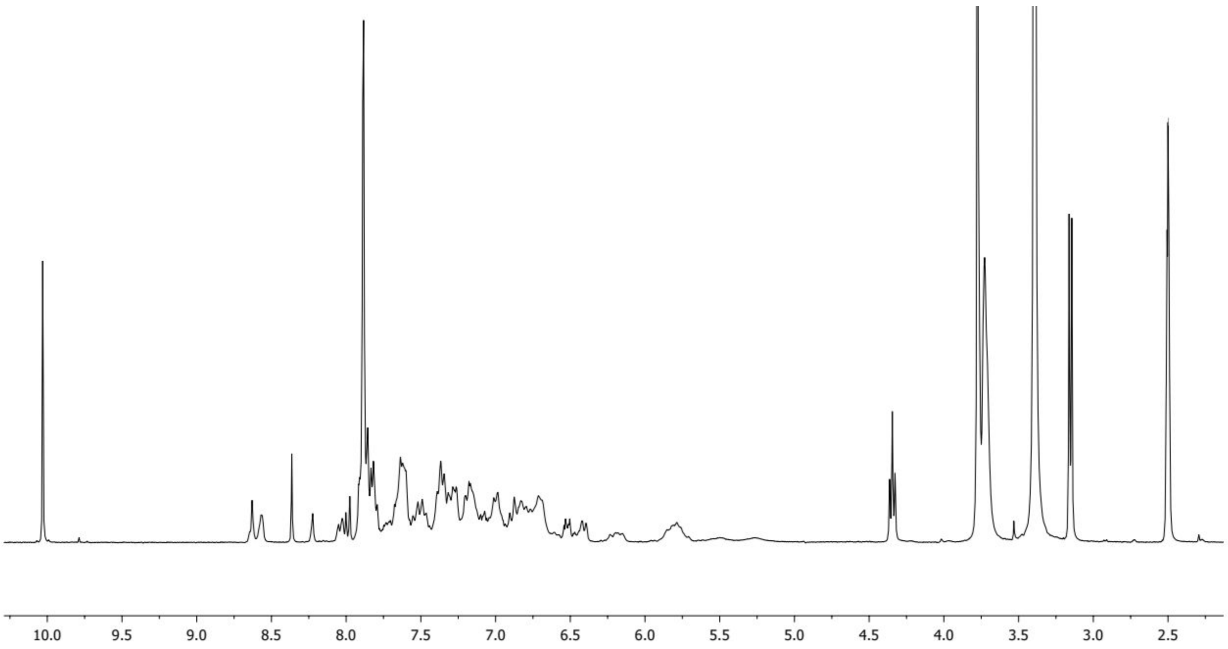

Figure S14.^{1}H NMR (300 MHz, DMSO-d_6, temperature: 25 ˚C) spectrum of dynamic library composed from **1**, **3** and **6.** Reaction components: 5 mM.

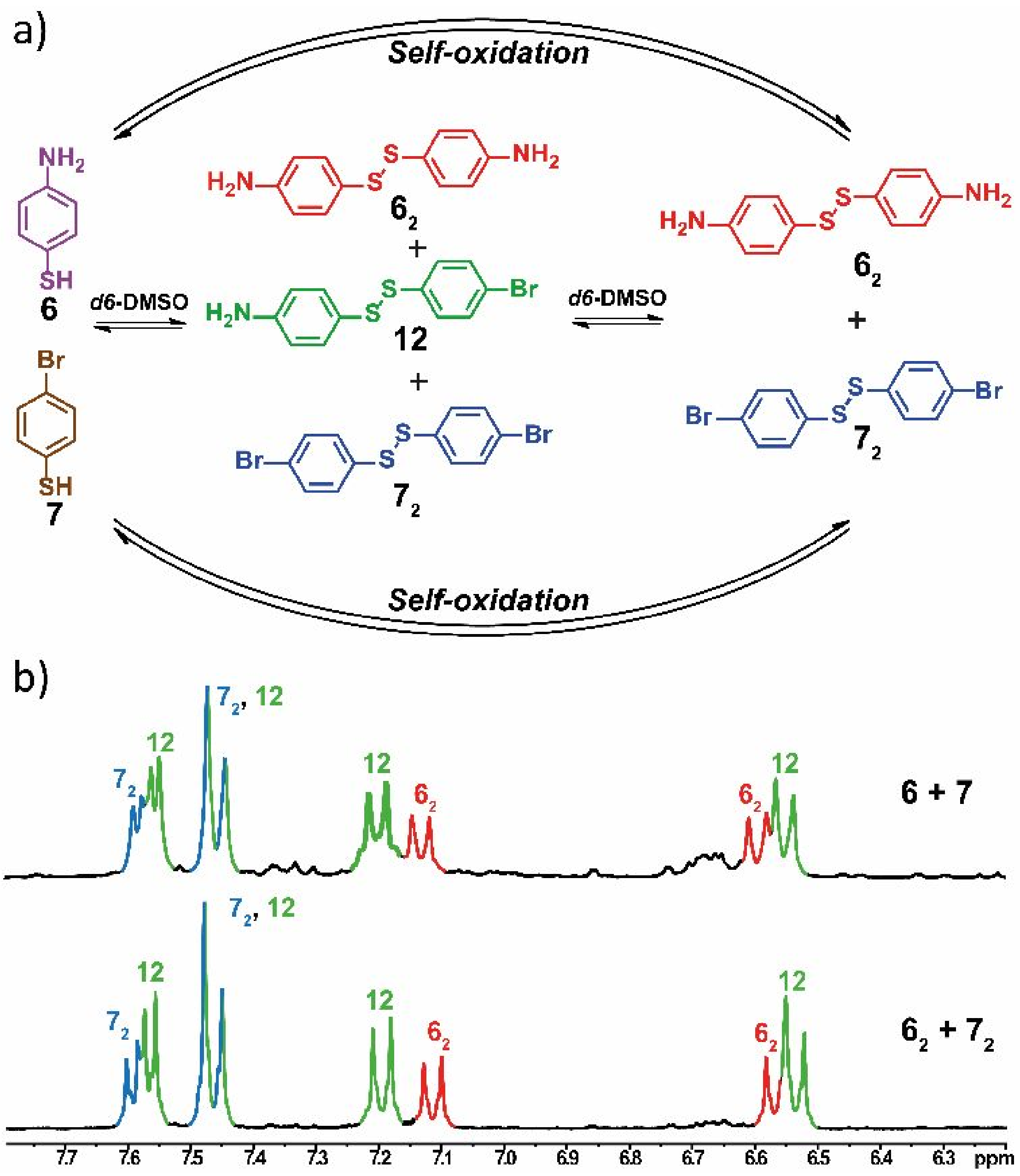

Figure S15. Determination of thermodynamic equilibrium between disulfide compounds. a) Reaction scheme presenting formation of disulfide library using two separate pathways; b) 1H NMR (300 MHz, DMSO-d_6, temperature: 25 °C) spectra of disulfide library formation: from thiol components **6** and **7** (top) and from preformed disulfides $\mathbf{6_2}$ and $\mathbf{7_2}$ (bottom). All reaction components at 5 mM.

IV XRD Data

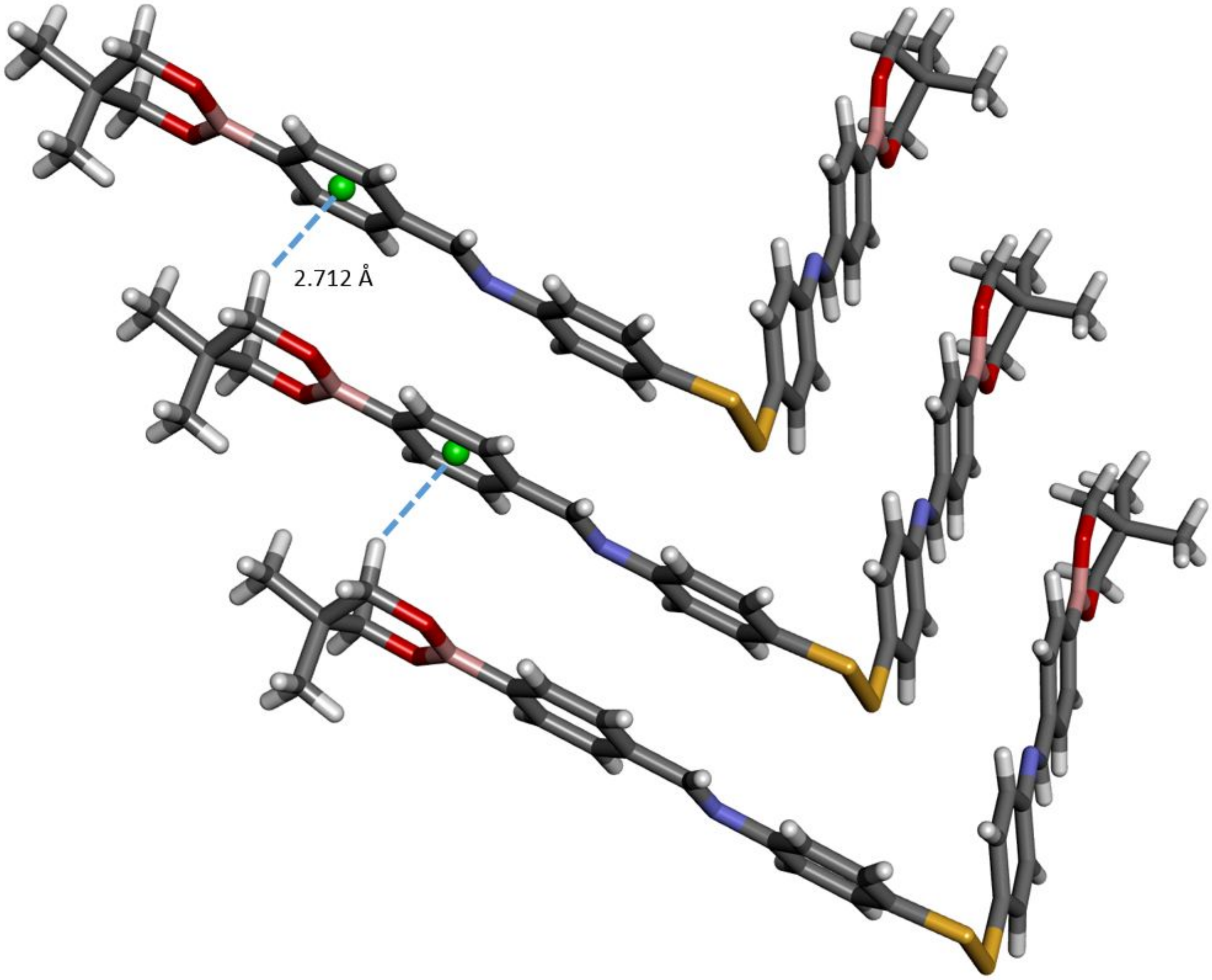

Figure S16. The weak CH···π interactions, established between the methylene hydrogen atom and the aromatic six-membered ring (CH···πPh 2.712(1) Å) of compound **16**.

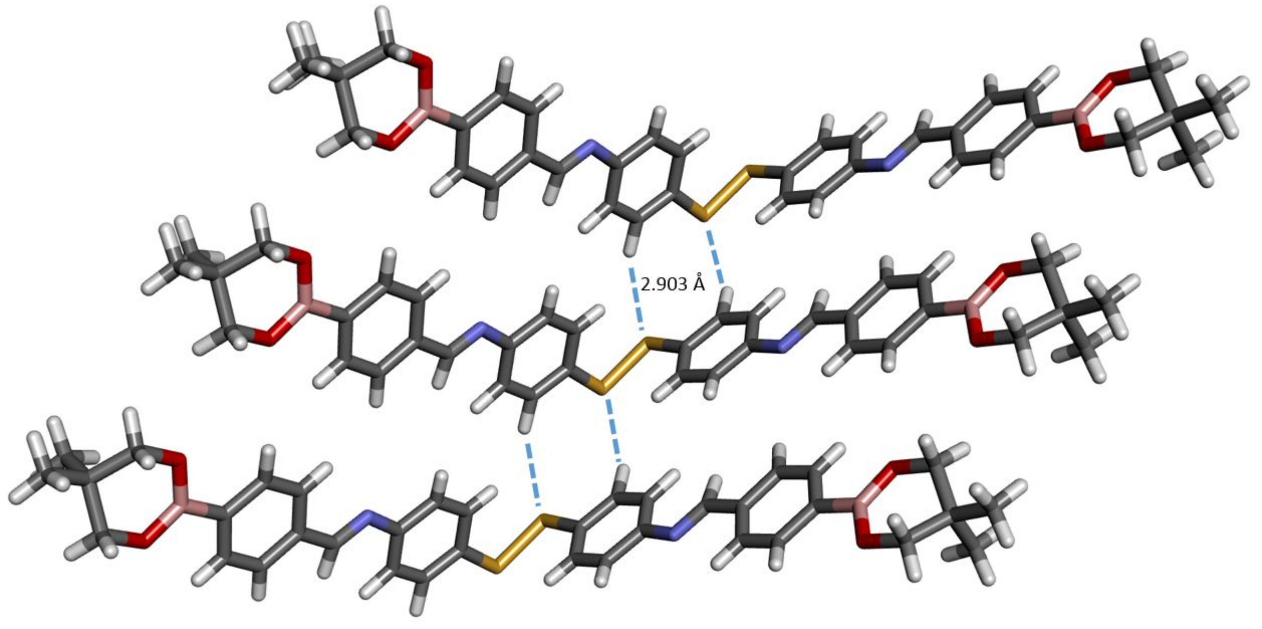

Figure S17. The weak S···CHPh interactions (2.903(1) Å) along to the *a* axis.

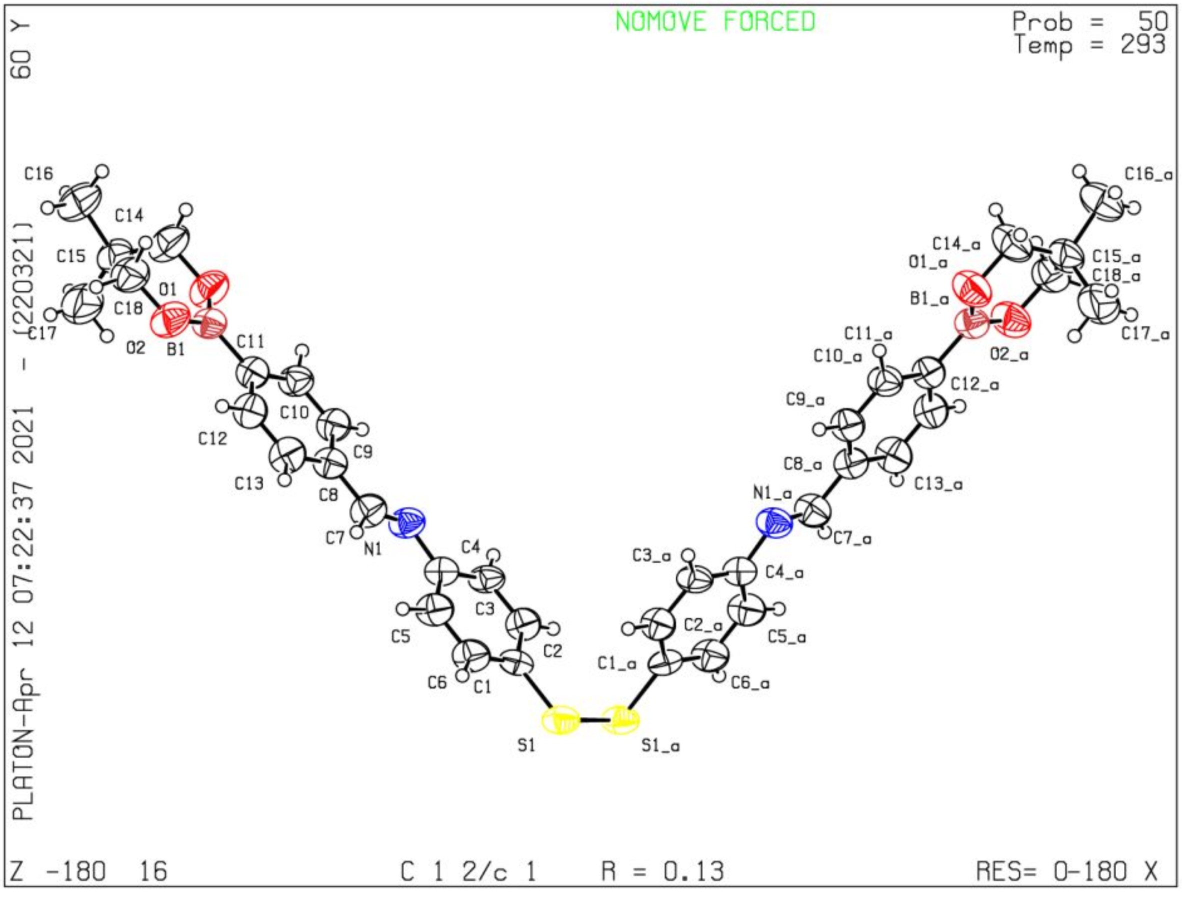

Figure S18. ORTEP representation of **16**. Displacement probability ellipsoids are shown at the 50% level.

Table S1. Crystal data and structure refinement for compound **16.**

Identification code	**16**
Empirical formula	$C_{36}H_{38}B_2N_2O_4S_2$
Formula weight	648.42
Temperature/K	293(2)
Crystal system	monoclinic
Space group	C2/c
a/Å	6.2895(8)
b/Å	10.7081(12)
c/Å	49.502(9)
α/°	90
β/°	94.544(14)
γ/°	90
Volume/Å^3	3323.4(8)
Z	4
ρ_{calc}g/cm^3	1.296
μ/mm^{-1}	0.203
F(000)	1368.0
Crystal size/mm^3	0.3 × 0.2 × 0.2
Radiation	MoKα (λ = 0.71073)
2Θ range for data collection/°	6.606 to 51.074
Index ranges	-7 ≤ h ≤ 7, -12 ≤ k ≤ 12, -58 ≤ l ≤ 58
Reflections collected	35769
Independent reflections	2928 [R_{int} = 0.1433, R_{sigma} = 0.0712]
Data/restraints/parameters	2928/0/210
Goodness-of-fit on F^2	1.119
Final R indexes [I>=2σ (I)]	R_1 = 0.1358, wR_2 = 0.3982
Final R indexes [all data]	R_1 = 0.1821, wR_2 = 0.4245
Largest diff. peak/hole / e Å^{-3}	0.95/-0.39

[1] CrysAlisPRO, *Oxford Diffraction., Agilent CrysAlis PRO. Agilent Technologies Ltd* **2014**, Yarnton, Oxfordshire, England.

[2] O. V. Dolomanov, L. J. Bourhis, R. J. Gildea, J. A. K. Howard, H. Puschmann, *J. Appl. Crystallogr.* **2009**, *42*, 339-341.

[3] G. Sheldrick, *Acta Crystallographica Section A* **2015**, *71*, 3-8.

[4] G. Sheldrick, *Acta Crystallographica Section C* **2015**, *71*, 3-8.
